# MoDAFold: a strategy for predicting the structure of missense mutant protein based on AlphaFold2 and molecular dynamics

**DOI:** 10.1093/bib/bbae006

**Published:** 2024-02-01

**Authors:** Lingyan Zheng, Shuiyang Shi, Xiuna Sun, Mingkun Lu, Yang Liao, Sisi Zhu, Hongning Zhang, Ziqi Pan, Pan Fang, Zhenyu Zeng, Honglin Li, Zhaorong Li, Weiwei Xue, Feng Zhu

**Affiliations:** College of Pharmaceutical Sciences, The Second Affiliated Hospital, Zhejiang University School of Medicine, Zhejiang University, Hangzhou 310058, China; Industry Solutions Research and Development, Alibaba Cloud Computing, Hangzhou 330110, China; College of Pharmaceutical Sciences, The Second Affiliated Hospital, Zhejiang University School of Medicine, Zhejiang University, Hangzhou 310058, China; College of Pharmaceutical Sciences, The Second Affiliated Hospital, Zhejiang University School of Medicine, Zhejiang University, Hangzhou 310058, China; Industry Solutions Research and Development, Alibaba Cloud Computing, Hangzhou 330110, China; College of Pharmaceutical Sciences, The Second Affiliated Hospital, Zhejiang University School of Medicine, Zhejiang University, Hangzhou 310058, China; Industry Solutions Research and Development, Alibaba Cloud Computing, Hangzhou 330110, China; College of Pharmaceutical Sciences, The Second Affiliated Hospital, Zhejiang University School of Medicine, Zhejiang University, Hangzhou 310058, China; Key Laboratory of Elemene Class Anti-cancer Chinese Medicines, School of Pharmacy, Hangzhou Normal University, Hangzhou 311121, China; College of Pharmaceutical Sciences, The Second Affiliated Hospital, Zhejiang University School of Medicine, Zhejiang University, Hangzhou 310058, China; College of Pharmaceutical Sciences, The Second Affiliated Hospital, Zhejiang University School of Medicine, Zhejiang University, Hangzhou 310058, China; Industry Solutions Research and Development, Alibaba Cloud Computing, Hangzhou 330110, China; Innovation Institute for Artificial Intelligence in Medicine of Zhejiang University, Alibaba-Zhejiang University Joint Research Center of Future Digital Healthcare, Hangzhou 330110, China; Industry Solutions Research and Development, Alibaba Cloud Computing, Hangzhou 330110, China; Innovation Institute for Artificial Intelligence in Medicine of Zhejiang University, Alibaba-Zhejiang University Joint Research Center of Future Digital Healthcare, Hangzhou 330110, China; School of Pharmacy, East China University of Science and Technology, Shanghai 200237, China; Industry Solutions Research and Development, Alibaba Cloud Computing, Hangzhou 330110, China; Innovation Institute for Artificial Intelligence in Medicine of Zhejiang University, Alibaba-Zhejiang University Joint Research Center of Future Digital Healthcare, Hangzhou 330110, China; School of Pharmaceutical Sciences, Chongqing University, Chongqing 401331, China; College of Pharmaceutical Sciences, The Second Affiliated Hospital, Zhejiang University School of Medicine, Zhejiang University, Hangzhou 310058, China; Industry Solutions Research and Development, Alibaba Cloud Computing, Hangzhou 330110, China; Innovation Institute for Artificial Intelligence in Medicine of Zhejiang University, Alibaba-Zhejiang University Joint Research Center of Future Digital Healthcare, Hangzhou 330110, China

**Keywords:** MoDAFold, missense mutant protein, protein structure prediction, deep learning, molecular dynamics

## Abstract

Protein structure prediction is a longstanding issue crucial for identifying new drug targets and providing a mechanistic understanding of protein functions. To enhance the progress in this field, a spectrum of computational methodologies has been cultivated. AlphaFold2 has exhibited exceptional precision in predicting wild-type protein structures, with performance exceeding that of other methods. However, predicting the structures of missense mutant proteins using AlphaFold2 remains challenging due to the intricate and substantial structural alterations caused by minor sequence variations in the mutant proteins. Molecular dynamics (MD) has been validated for precisely capturing changes in amino acid interactions attributed to protein mutations. Therefore, for the first time, a strategy entitled ‘*MoDAFold*’ was proposed to improve the accuracy and reliability of missense mutant protein structure prediction by combining AlphaFold2 with MD. Multiple case studies have confirmed the superior performance of *MoDAFold* compared to other methods, particularly AlphaFold2.

## INTRODUCTION

Protein structure prediction has been one of the longstanding issues, which is crucial for uncovering novel drug targets and facilitating a mechanistic understanding of protein functions [1–3]. With the advancement of next-generation sequencing, large amounts of protein sequences have accumulated, and over 200 million arrangements have been available in UniProt [4]. Acquiring experimentally validated protein structures is considerably more challenging compared to protein sequences, primarily due to its time-consuming and labor-intensive nature. [5–7]. So far, the *RCSB Protein Data Bank* (PDB) includes only 200 thousand protein structures [8], which asks for the development of new strategies to significantly accelerate the process of protein structure prediction [9–11]. Thus, a variety of computational methods have been constructed to facilitate the research developments in this particular direction [12–14], which successfully promotes the identification of efficacy drug targets, the understanding of the molecular mechanism underlying protein functions and so on [15–17].

However, the longstanding challenges for protein structure prediction based on computational methods are insufficient awareness of protein structure prediction on the ground of sequences [18] and the sophistication of protein folding processes [19, 20]. Notably, missense mutations in proteins have the potential to significantly perturb the folding free energy of mutant proteins compared to their wild-type (WT) counterparts [21, 22]. This distinction has been reported to change the protein folding process, making the prediction accuracies of existing methods hardly satisfactory [23–25]. In other words, it is still extremely challenging for current methods/tools to improve the prediction accuracy for mutant protein structures, and it is essential to develop methods for protein structure prediction. To address this critical issue, two distinct computational strategies have been proposed, broadly categorized as homology modeling (HM)-based methods [26–28] and machine learning (ML)-based ones [29–31].

HM-based strategy has been widely used for protein structure prediction, and many tools have been developed (HOMELETTE, SWISS-MODEL, GPCRM) [26–28], but they are severely dependent on the homology among the analyzed sequences. To deal with this issue, ML-based strategy has thus been constructed, which learns protein structures irrespective of sequence homology [29–31]. Some typical tools under this strategy include AlphaFold2, ColabFold and RoseTTAFold, all of which apply machine learning framework(s) to achieve great predictive performance. For instance, the AlphaFold2 in ‘*The 14th Critical Assessment of Protein Structure*’ *Prediction* (CASP14) [12], showcased a level of accuracy that competes with experimental structures, significantly surpassing the performance of other methods. However, due to the dramatic effect of missense mutation on the protein folding process and the fact that training data for AlphaFold2 do not contain altered structures of these mutated proteins [32–34], the performance of AlphaFold2 for predicting missense mutations on protein structures is severely decreased. This limitation of AlphaFold2 was also reported in the journal *Nature Structural & Molecular Biology* and the FAQ on the AlphaFold2 Protein Database website [35, 36]. Besides, since most methods predict structures based on those available in the PDB rather than by driving forces of protein folding [36], it is still extremely challenging for existing methods to improve the prediction performance of mutant protein structures.

Herein, a protein structure prediction strategy combining AlphaFold2 with molecular dynamics (MD), named ‘*MoDAFold*’ was proved to improve the accuracy and reliability of mutant protein structure prediction. *First*, leveraging the approach for protein structure prediction, AlphaFold2 [37–39], we conducted predictions of both WT and mutant structures based on the corresponding protein sequences. *Second*, *Assisted Model Building with Energy Refinement* (Amber, a typical tool for MD) [40, 41] was employed to refine the predicted protein structures derived from AlphaFold2. *Finally*, six protein structures with significant pair distinctions between WT and mutant protein underlying only a single mutation were collected to evaluate the performance of this new strategy (MoDAFold). The prediction structures of these WT and mutant proteins predicted by AlphaFold2/MoDAFold were aligned with those experimental structures and evaluated by the *root-mean-square deviation* (RMSD) metric [42, 43], respectively. All in all, our MoDAFold was expected to significantly improve the performance of mutant protein structure prediction. Multiple case studies based on benchmark were also conducted, which confirmed the superior performance of MoDAFold than AlphaFold2 [36].

## RESULTS

### Formation of pocket (channel) in BRCA1-BRCT induced by A1708E mutation

The *Breast cancer type 1 susceptibility protein* (BRCA1) is an essential mediator protein in DNA damage-induced nuclear signaling events [44, 45]. The BRCT domain is a tandem pair of repeats at the BRCA1 C-terminal region, which mediates the interactions with phosphorylated partner proteins, such as DNA helicase, BACH1, etc. [46, 47]. There is an important missense mutation on BRCT, A1708E, which is closely associated with an increased risk of breast and ovarian cancer [48]. As reported, A1708 is wrapped in a small hydrophobic pocket between two BRCT repeats, and replacement with the bulkier and charged glutamic acid residue is expected to destabilize their interaction [36]. Moreover, the A178E missense variant at this position strongly disrupts the interaction of BRCT with phosphopeptides and the stability of the protein fold. In other words, the A1708E mutation enlarges the hydrophobic pocket of the mutated position in the BRCA1-BRCT, which has a significant impact on changing its structure and function, leading to distinctive structural differences from the WT [49]. Thus, to address the great impact of missense mutations on protein structure alterations, protein structures of WT and mutant protein structures (A1708E) were predicted in this study using AlphaFold2. The corresponding systems simulated with Amber, and the RMSD among the related forms were calculated by PyMOL [50].

**Figure 1:**
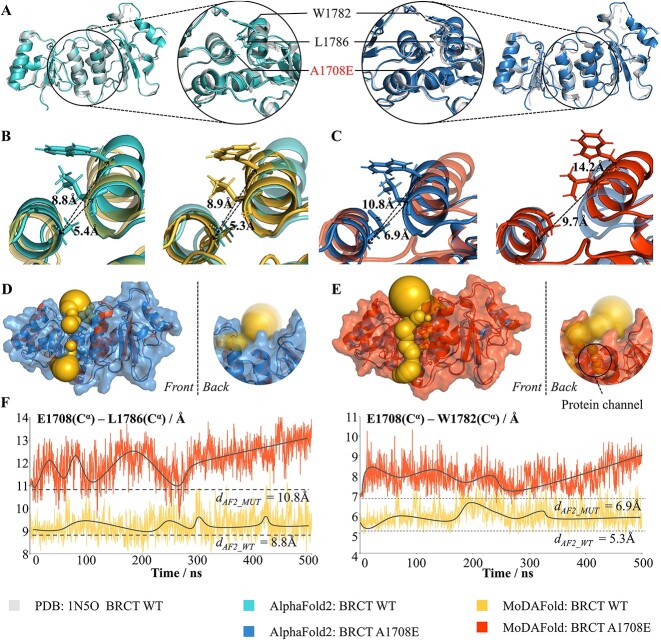
Structural prediction for mutant and WT BRCT by AlphaFold2 and MoDAFold. (**A**) Overlayed the experimental BRCT structure (grey, PDB ID: 1N5O) and AlphaFold2 predicted structure for WT BRCT (light blue, left). Overlayed the experimental BRCT structure (grey) and AlphaFold2 predicted structure for A1708E (blue, right). Sidechain-heavy atoms are displayed for position 1708 and the surrounding residues. b, Overlayed AlphaFold2 predicted structure (light blue, left) and MoDAFold simulated structure (yellow, right) for WT BRCT. Distances of E1708-L1786 and E1708-W1782 Cα don’t increase after the dynamics simulation. (**C**) Overlayed AlphaFold2 predicted structure (blue ,left) and MoDAFold simulated structure (orange, right) for BRCT A1708E. Distances of E1708-L1786 and E1708-W1782 Cα increase a lot after the dynamics simulation. (**D**) The surface (blue) and cavity (yellow ball) of BRCT A1708E structure predicted by AlphaFold2. (**E**) The surface (orange) and cavity (yellow ball) of BRCT A1708E structure predicted by MoDAFold. The black oval highlights the protein channel surrounding position 1708 in the mutant. (**F**) Trends in E1708-L1786 and E1708-W1782 Cα distances of WT (yellow, above) and A1708E (orange, below) during MD simulations.

AlphaFold2 predicted glutamic acid-substituted BRCT at position 1708 (Figure 1A; right, blue) to be structurally equivalent to WT BRCT (Figure 1A; left, light blue) with only minor differences in RMSD, which were 0.81 and 0.53 Å compared to the experimental structure (grey), respectively. Additionally, for A1708E BRCT, there is slightly more space between the helices of the two repeats (Figure 1B, C), with the distance between the α-carbons of residues 1708 and 1782 at 5.4 Å for WT (Figure 1B; left) and 6.9 Å for the A1708E mutant (Figure 1C; left). The E1708(Cα)–W1786(Cα) distances at 8.8 Å for WT (Figure 1B; left) and 10.8 Å for the A1708E mutant (Figure 1C; left). This increased distance to accommodate the longer glutamic acid was insufficient to prevent the interaction of the acidic amino acid E1708 with the hydrophobic amino acid L1786. Generally, this study confirms that AlphaFold2 cannot accurately predict the protein structure of missense mutations (A1708E) in BRCT as illustrated by previous reports [36].

While MoDAFold performed comparably to AlphaFold2 in predicting the WT structure, it showed better performance in predicting the mutant structure under the evaluation criteria (Cα distance, space-filling of hydrophobic pockets). Specifically, the E1708(Cα)–W1782(Cα) distances were 5.3 Å for WT BRCT (Figure 1B; right), 9.7 Å for A1708E BRCT (Figure 1C; right), and the E1708(Cα)–L1786(Cα) distances were 8.9 Å for WT BRCT (Figure 1B; right), 14.2 Å for A1708E BRCT (Figure 1C; right). Due to the increasing distances among three amino acids leading to the enlargement of the hydrophobic pocket between two BRCT repeats, the pocket of the simulated A1708E mutant (orange) was significantly larger than that of the WT (blue) by hydrophobic pockets filled with yellow spheres, respectively (Figure 1D, E). Surprisingly, a pocket(channel) formation of pocket (channel) in BRCA1-BRCT was induced by A1708E mutation, and this channel was not predicted by AlphaFold2. The changes in WT and mutant structures during MD simulation were also displayed by the trend of the distances among E1708(Cα), W1782(Cα) and L1786(Cα). Furthermore, the E1708(Cα)–L1786(Cα) and the E1708(Cα)–W1782(Cα) distances of the A1708E mutant increased after 300 ns, and these of WT were smooth during the 500 ns MD simulation. The result indicates that MoDAFold could predict the trend in structural changes of A1708E BRCT. Two other mutant proteins mentioned by *Nature Structural & Molecular Biology* () were also studied similarly, and the predicted structure of the mutant proteins was also somewhat enhanced (more details were described in Supplementary information 1 and 2).

### Unfolding of engrailed homeodomain induced by L16A mutation

**Figure 2:**
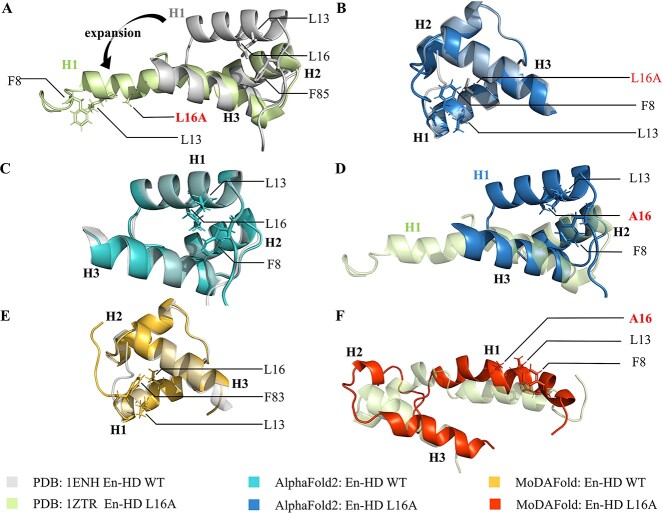
Structural prediction for mutant and WT En-HD by AlphaFold2 and MoDAFold. (**A**) Overlayed the experimental En-HD structure (grey, PDB ID: 1ENH) and En-HD L16A structure (light green, PDB ID: 1ZTR). Sidechain-heavy atoms are displayed for position 16 and the surrounding residues. The arrow indicates the expansion of helix 1. (**B**) Overlayed the experimental En-HD structure (grey) and AlphaFold2 predicted structure for En-HD L16A (blue). (**C**) Overlayed the experimental En-HD structure (grey) and AlphaFold2 predicted structure for WT En-HD (light blue). (**D**) Overlayed the experimental En-HD L16A structure (light green) and AlphaFold2 predicted structure for En-HD L16A (blue). (**E**) Overlayed the experimental En-HD structure (grey) and WT En-HD structure predicted by MoDAFold (yellow). (**F**) Overlayed the experimental EnHD L16A structure (light green) and En-HD L16A structure predicted by MoDAFold (orange).

Homeodomains are common eukaryotic DNA-binding domains that consist of a short-extended strand with 3 helices [51, 52]. *Drosophila melanogaster* Engrailed homeodomain (En-HD) is a 61-residue three-helix bundle protein with helices spanning residues 10–22 (H1), 28–37 (H2) and 42–56 (H3) [53]. The L16A mutation of En-HD eliminates several local interactions and numerous long-range interactions with residues in H2, H3, and the turn between H2 and H3 that leads to the unfolding of the helix 1 (Figure 2A) [54]. Meanwhile, multiple conformers of the solution structure of Engrailed homeodomain L16A mutant are provided in the PDB database, and the different conformations of H1 (helix 1) are a consequence of the lack of a significant number of long-range NOEs between them and residues 28–53 (Protein Data Bank code 1ZTR).

AlphaFold2 predicts similar structures for WT and L16A En-HD, with an average RMSD of only 0.49 Å. The mean pLDDT score of L16A mutation is 87.7, which is lower than that of the top-ranking WT structure, with a 94.1 score (Figure 2B, C). The RMSD is about 11.1 Å between 1ZTR (solution structure supported by PDB database) and AlphaFold2 predicting the structure of Engrailed homeodomain L16A mutant (Figure 2D). After performing MD simulations at constant pH 5.7, the helix1 is unfolded, and the relative positions of the three helices in the predicted overall structure are consistent with the actual solution structure, with an average RMSD. of 6.7 Å (the H1 helix is not fixed in the solution structure) (Figure 2F).

### Structural rearrangements of prion protein induced by V210I mutation

**Figure 3:**
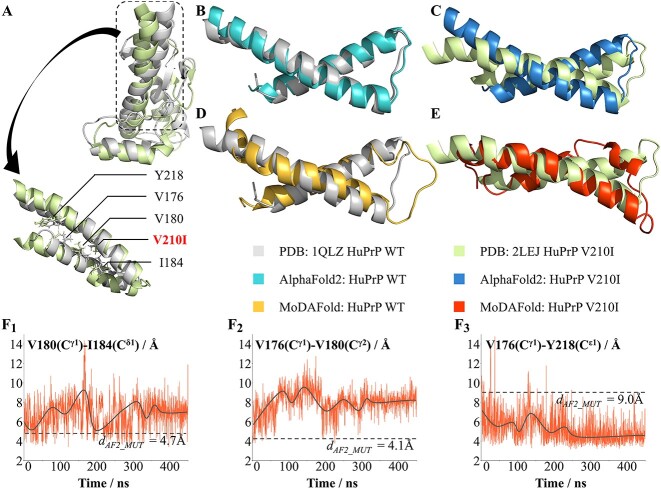
Structural prediction for mutant and WT HuPrP by AlphaFold2 and MoDAFold. (**A**) Overlayed the experimental HuPrP structure (grey, PDB ID: 1QLZ) and HuPrP V210I structure (light green, PDB ID: 2LEJ). The black rectangle highlights the α2 and α3 helices of experimental HuPrP (grey) and HuPrP V210I (light green). Sidechain-heavy atoms are displayed for position 210 and the surrounding residues. (**B**) Overlayed the α2 and α3 helices of experimental HuPrP (grey) and AlphaFold2 predicted structure for HuPrP (light blue). (**C**) Overlayed the α2 and α3 helices of experimental HuPrP V210I (light green) and AlphaFold2 predicted structure for HuPrP V210I (blue). (**D**) Overlayed the experimental HuPrP structure (grey) and HuPrP structure predicted by MoDAFold (yellow). (**E**) Overlayed the experimental HuPrP V210I structure (light green) and HuPrP V210I structure predicted by MoDAFold (orange). (**F**) Trends in V180(Cγ1)–I184(Cδ1), V176(Cγ1)–V180(Cγ2), V176(Cγ1)–Y218(C ɛ1) distances of HuPrP V210I during MD simulations.

Prion protein is closely related to transmissible spongiform encephalopathies, which are deadly diseases and the NMR structures of human prion protein ((HuPrP)) contain a globular domain with three α-helices and a short anti-parallel β-sheet (Figure 3A) [55, 56]. Comparison with the structure of the WT (PDB ID: 1QLZ) revealed that although the two structures share similar global architecture, its V210I mutation (PDB ID: 2LEJ) introduces some local structural differences. The variations reported are mainly concentrated in the α2–α3 inter-helical interface and in the β2–α2 loop region (Figure 3A), as residue 210 is part of a hydrophobic core that is critical to the overall stability of the protein [57, 58]. The residues 180 and 210 are located in the α2–α3 helix interface on WT, which is associated with direct hydrophobic contacts. After mutation, presumably due to steric crowding, the side chain of Val180 changes direction. Furthermore, the side chains of the other two residues, Val176 and Ile184, are also significantly shifted compared to their positions in the WT protein. Thus, these rearrangements affect several hydrophobic contacts commonly present in WT proteins, especially residue Val180. This change can be seen visually by the different distances among these amino acids from WT (Supplementary Table S1). Another significant structural variation in comparison to the WT is the β2–α2 loop region showed by Tyr169–Phe175, Phe175–Tyr218, Tyr163–Tyr218 and Tyr163–Phe175 distances (Supplementary Table S2) [59].

However, AlphaFold2 cannot capture the small structural changes brought about by this mutation. The structure predicted by AlphaFold2 of V210I mutation is almost identical to the expected WT structure with an average RMSD of only 1.5 Å, while very different from the actual mutant structure with an average RMSD of 2.6 Å (Figure 3B, C). According to the experimental information provided by the PDB database, the relative positions of the α2–α3 region in the mutant protein are closer to the actual mutant structure reported after a period of simulation in the solution environment of pH 5.5 (Figure 3E), shown by intra- and inter-helical distances between residues from α2 and α3 helices (Figure 3F; Supplementary Table S1). In contrast, the WT structure did not change much after the simulation for a while (Figure 3D). Likewise, the protein structure after dynamics simulation is closer to the actual crystal structure than that predicted by AlphaFold2 from distances between residues involved in the interface of β2, α2 and α3 secondary structure elements (Supplementary Table S2).

### Fold switching of protein G induced by L45Y mutation

**Figure 4:**
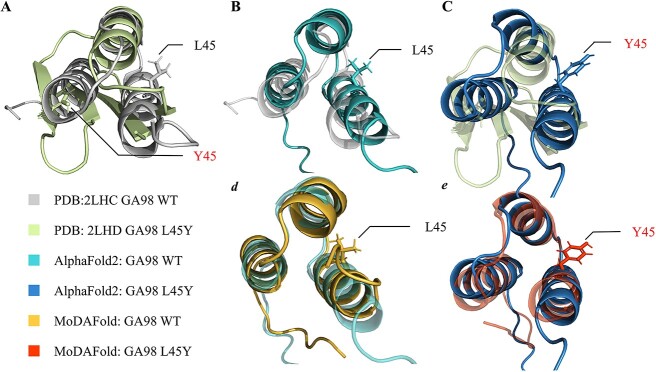
Structural prediction for mutant and WT GA98 by AlphaFold2 and MoDAFold. (**A**) Overlayed the experimental GA98 structure (grey, PDB ID: 2LHC) and GA98 L45Y structure (light green, PDB ID: 2LHD). Sidechain-heavy atoms are displayed for position 1708. (**B**) Overlayed the experimental GA98 structure (grey) and AlphaFold2 predicted structure for GA98 (light blue). (**C**) Overlayed the experimental GA98 L45Y (light green) and AlphaFold2 predicted structure for GA98 L45Y (blue). (**D**) Overlayed AlphaFold2 predicted structure (light blue) and MoDAFold simulated structure (yellow) for WT GA98. (**E**) Overlayed AlphaFold2 predicted structure (blue) and MoDAFold simulated structure (orange) for GA98 L45Y.

While disorder-to-order rearrangements are relatively common, the ability of proteins to switch from one ordered fold to an entirely different fold is generally considered rare and few fold switches have been characterized [60, 61]. However, the GA domain adopts a 3-α helix bundle structure (PDB ID: 2LHC) and binds human serum albumin. In contrast, the GB domain with only one mutation L45Y, has a 4β + α fold (PDB ID: 2LHD) and binds immunoglobulin G (IgG) (Figure 4A) [62, 63]. So, the A and B domains of protein G are classic model systems of folding for decades, the subject of numerous experimental and computational studies. The study has investigated the folding of this protein by using a Markov State Model (MSM) built on about 50 ms of MD simulations, and models such as the one presented have been successful at comparing with experiments and providing atomic-level detail of folding reactions [64].

However, after 500 ns of ordinary dynamics simulation and 1.5 μs of accelerated dynamics simulation, the improvement effect of MD on other proteins, which are 56-amino-acid domains termed GA and GB from the multi-domain *Streptococcus* cell surface protein G [65], is fragile. The structure of GB 98 predicted by AlphaFold2 (Figure 4C) is similar to GA 98 indicated (Figure 4B), and it is shown that MD cannot correct erroneous structure for single point mutations with significant changes in secondary structure by its simulating results (Figure 4D, E).

## DISCUSSION AND CONCLUSION

AlphaFold2 has limitations in accurately describing protein structures for missense mutant proteins [36], which are described in previous studies and also confirmed by our study. The limit arises from the fact that AlphaFold2’s predictions are based on known sequence and structure data rather than the physical laws of protein folding, and its training data does not include altered structures of mutant proteins [66]. Therefore, combining AlphaFold2 with physics-based computational methods like MD simulations can be a valuable strategy for accurately predicting the structure of point mutant proteins.

This study finds that in some cases where missense mutations only affect the positions between secondary structures, MD simulations can provide predictions after a simulation period [67]. However, the initial structure used in simulations may be unreasonable for mutants with significant changes in secondary structure. Unique simulation methods (such as MSM [68]) or long-time simulations can be employed to simulate these challenging protein structures successfully. The accuracy of simulations generally depends on the availability of accurate experimental protein structures or reliable homology models as initial conditions. Using protein structures predicted by AlphaFold2 as initial structures for MD simulations can improve the accuracy of predicting the structures of missense mutant proteins. However, improvements in the model or the use of other protein secondary structure prediction methods may be necessary for mutants with significantly altered secondary structures.

In conclusion, the study suggests that the tertiary structures of mutant proteins predicted by AlphaFold2 need to be more accurate. MoDAFold, which combines AlphaFold2 with MD simulations, has shown superior performance in predicting the structures of missense mutant proteins in multiple cases. MoDAFold is expected to significantly enhance the accuracy of mutant protein structure prediction and represents an important strategy for accurately predicting the structures of missense mutant proteins.

## MATERIALS AND METHODS

### WT and mutant proteins data collection

In this study, six protein structure pairs with significant distinctions between WT and mutant protein underlying only a single mutation were collected to evaluate the performance of this new strategy (MoDAFold). Three proteins (BRCT, MyUb and UBAs) were applied to discuss whether AlphaFold2 can predict the impact of missense mutations on structure [36], and they were also adopted in our study. The experimental mutant structures of these three proteins were unavailable in PDB, so the other three proteins with mutant structures in PDB were selected by following the pipeline as shown in Supplementary Figure S1. *First*, more than 16,000 papers related to missense mutant proteins were scanned and 54 related missense single-nucleotide variants were screened from these papers (as shown in Supplementary Table S1). *Second*, only fourteen pairs of proteins whose WT and mutant proteins had experimentally solved structures in PDB were selected for the subsequent analysis. *Finally*, we selected proteins with RMSD greater than 2 Å, and to prevent the interaction between the strands from affecting the results, we chose single-stranded proteins. Three pairs of proteins (En-HD, HuPrP and GA98) were chosen for prediction to compare the prediction effects of these methods intuitively. *As a result*, six pairs of proteins were collected for structure prediction of WT and mutant proteins and performance comparison of methods.

### Structure prediction

#### Protein structure prediction with AlphaFold2

The first step of our strategy (*MoDAFold*) was to predict the WT and mutant structures based on protein sequences using AlphaFold2. By introducing ‘Evoformer’ module, combined with multiple sequence alignments and equivariant attention architecture, AlphaFold2 achieves accurate prediction of the 3D coordinates of all heavy atoms of a give protein sequence. It also provides the predicted local-distance difference test score (pLDDT) of the corresponding structure, which is used to evaluate the performance of the structure prediction on a scale of 0–100, with closer to 100 indicating the better the prediction performance [12]. The previously collected WT and mutant protein sequences can be imported into the AlphaFold2 model to obtain the corresponding PDB files. The default parameters were used in the predicting process. It is important to note that protein prediction with AlphaFold2 is computationally intensive, and our server has a lot of CPUs and eight GPUs to support the computational consumption.

#### MD simulation with Amber

In this study, the state-of-the-art MD simulation (Gaussian accelerated molecular dynamics, GaMD) [69] is executed for six pairs of proteins screened including WT and mutant, using the structures predicted by AlphaFold2 as initial structures. During the experiment, most proteins are simulated in an aqueous solution, using sodium and chloride ions to balance the charge, but some proteins, such as the engrailed homeodomain and human prion protein, are simulated in a specific pH environment to approximate the experimental results. Besides, 10 Å of water was added per side to avoid protein–protein interactions. In addition, to ensure the stability of the obtained protein structure, GaMD [70] of 1 μs is performed after the long-term ordinary molecular simulation (more detail was shown in the Supplementary Method).

### Performance evaluation

The RMSD was applied in this study to evaluate the performance for protein structure prediction, which was a wild measurement to calculate distances between corresponding alpha-carbon atoms (C_α_) in two compared structures. The formula was as follows:


$$ RMSD=\sqrt{\frac{1}{n}{\sum}_{i=1}^n{\left({y}_i-\hat{y_i}\right)}^2} $$

where *n* represented the length of a protein sequence, and *i* denoted the position identifier for one amino acid in this protein. ${y}_i$and $\hat{y_i}$ indicated the C_α_ coordinates of the *i*th amino acid in two compared structures within 3D space, respectively. A smaller RMSD value means a lower difference between the experimental structure and the prediction. Meanwhile, these RMSD changes during the MD were was also used to monitor whether the simulation has reached a balance and to determine the final stable protein conformation.

**Key Points**

- MoDAFold was proposed to improve the accuracy and reliability of missense mutant protein structure prediction.
- MoDAFold combined AlphaFold2 and molecular dynamics (MD) to leverage the strengths of both methods.
- Multiple case studies have demonstrated the superior performance of MoDAFold compared to other methods, including AlphaFold2.

## Supplementary Material

MoDAFold_Supplementary_bbae006

## Data Availability

All data and codes were freely available to all users at https://github.com/idrblab/MoDAFold.git.
