# Supplementary material for: MoDAFold: a strategy for predicting the structure of missense mutant protein based on AlphaFold2 and molecular dynamics
Source: Brief Bioinform. 2024 Feb 1;25(2):bbae006. doi: 10.1093/bib/bbae006 (PMC10835750; doi:10.1093/bib/bbae006)
Supplement: MoDAFold_Supplementary_bbae006 [file modafold_supplementary_bbae006.docx]

**Supplementary Information for:**

**MoDAFold: a strategy for predicting the structure of missense mutant protein based on AlphaFold2 and molecular dynamics**

Lingyan Zheng**^1^**^,^**^2^**^,^†, Shuiyang Shi**^1^**^,^†, Xiuna Sun**^1^**^,^**^2^**†, Mingkun Lu**^1^**^,^**^2^**, Yang Liao**^1^**, Sisi Zhu**^3^**, Hongning Zhang**^1^**, Ziqi Pan**^1^**, Pan Fang**^2^**^,^**^4^**, Zhenyu Zeng**^2^**^,^**^4^**, Honglin Li**^5^**, Zhaorong Li**^2^**^,^**^4^**^,^*, Weiwei Xue**^6^**^,^*, Feng Zhu**^1^**^,^**^2^**^,^**^4^**^,^*

**^1^** College of Pharmaceutical Sciences, The Second Affiliated Hospital, Zhejiang University School of Medicine, Zhejiang University, Hangzhou, 310058, China

**^2^** Industry Solutions Research and Development, Alibaba Cloud Computing, Hangzhou, 330110, China

**^3^** Key Laboratory of Elemene Class Anti-cancer Chinese Medicines, School of Pharmacy, Hangzhou Normal University, Hangzhou 311121, China

**^4^** Innovation Institute for Artificial Intelligence in Medicine of Zhejiang University, Alibaba-Zhejiang University Joint Research Center of Future Digital Healthcare, Hangzhou, 330110, China

**^5^** School of Pharmacy, East China University of Science and Technology, Shanghai, 200237, China

**^6^** School of Pharmaceutical Sciences, Chongqing University, Chongqing 401331, China

* To whom correspondence should be addressed. Prof. Feng Zhu ([zhufeng@zju.edu.cn](mailto:zhufeng@zju.edu.cn)); Prof. Weiwei Xue (xueww@cqu.edu.cn); Mr. Zhaorong Li (zhaorong.lzr@alibaba-inc.com)

† These authors contributed equally to this work as co-first authors.

**Supplementary Information**

**1. Misfolding of MyUb induced by R1117A mutation**

*Myosin VI Ubiquitin-binding domain* (MyUb) is a helix-turn-helix-like structure domain that is critical for cargo trafficking and sorting during early endocytosis and autophagosome maturation [1, 2]. Especially, amino acid substitution of this motif with the alanine triple (AAA) is reported to abolish myosin VI interaction with these autophagy adaptor proteins [3]. MyUb (G1080–H1122) includes an RRL motif that resides in at C-terminal helix (Helix2) where R1117 forms hydrogen bonds to S1087 and E1113, so the replacement at this position from arginine to alanine acid is expected to result in misfolding and abolished interaction with ubiquitin [4]. In other words, R1117 forms hydrogen bonds across the MyUb structure, bridging the two helices, the R1117A mutation may therefore lead to the change in the relative position of the two helixes. In this study, the MyUb structures of the wild-type and the mutant (R1117A) proteins were predicted by AlphaFold2 and MoDAFold. The final structures were displayed and the RMSD among those structures were calculated with PyMOL [5].

AlphaFold2 predicted WT MyUb (**Fig. 2a**; light blue) to be structurally equivalent to experimental structure (**Fig. 2a**; grey) with 1.64 Å RMSD. While R1117A MyUb predicted by AlphaFold2 (**Fig. 2b**; light blue) is even more similar architecture with only 0.63 RMSD compared to experimental structure (**Fig. 2b**; grey). However, the distances among three amino acids (S1087, E1113 and R1117) on MyUb were close enough and there were hydrogen bonds among them. Alanine substitution at position 1708 causes the disappearance of the hydrogen bonds between two helixes (Fig. 2b), but AlphaFold2 did not capture this change. In other words, AlphaFold2 could not predict the change in MyUb structures due to the R1117A mutation. MoDAFold performed comparably to AlphaFold2 in predicting the WT structure. Particularly, due to the hydrogen bonds among three amino acids (S1087, E1113 and R1117), the WT structure which was stimulated by Amber after 1.5 μs (**Fig. 2c**; yellow) had little change in the relative position of the two helixes compared to AlphaFold2 (**Fig. 2c**; light blue). While for the R1117A MyUb, there was a relative movement between two helices of the R1117A mutant MyUb after 1.5 μs stimulation under the same conditions. While for the R1117A MyUb (**Fig. 2d**; orange) predicted by MoDAFold, there was a relative movement between two helices of the R1117A mutant MyUb after 1.5 μs stimulation under the same conditions, due to the loss of hydrogen bonds, which results in a significant change in the distance between two helices compared to AlphaFold2 (**Fig. 2d**; blue ,as shown by arrows) All in all, MoDAFold combined AlphaFold2 with MD performed better in predicting the relative movement between the two helixes of MyUb due to the R1117A mutant compared to AlphaFold2.

**2. Intrinsic disorder of ubiquitin-associated domain induced by L198A mutation**

*Ubiquitin-like modifier-activating enzyme 1* (UBA1) is one of the receptors for ubiquitin which is a prominent regulatory protein in numerous biological processes, such as catalytic ubiquitin conjugation, DNA repairs, and so on [6, 7]. There are three helixes in the structure of UBA1 and the L198 buried in the core of UBA1 is essential for its structural integrity [8, 9]. Therefore, the mutation at this position from leucine to alanine acid causes the UBA1 to become intrinsically disordered [9]. In other words, the L198A mutant structure of UBA1 has a large structural change compared with the wild-type protein structure, which destroys binding to ubiquitin. In this study, the UBA1 structures of the wild-type and the mutant (L198A) proteins were predicted by AlphaFold2 and MoDAFold. The final structures was displayed and the RMSD among those structures were calculated with PyMOL [5].

The accuracy of AlphaFold2 in predicting the structure of wild-type UBA1 was high, with an RMSD of 1.00 Å between the predicted (light blue) and experimental (grey) structures (**Fig. 3a**). In contrast, for the L198A mutation that induces disorder in the structure, the predicted structure of this mutant protein by AlphaFold2 (blue) exhibited only minor differences with an RMSD of 1.26 compared to the experimental wild-type structure (grey) (**Fig. 3b**). Remarkably, AlphaFold2 predicted the alanine-substituted UBA1 to be structurally equivalent to the wild-type UBA1, despite the arginine at position 179 moving towards the location occupied by L198 in the wild-type sequence (as indicated by the arrow in **Fig. 3b**). The insensitivity of AlphaFold2 to the effects caused by substituting the longer leucine sidechain with alanine was also evident from the similar mean pLDDT scores (84) for both structures. There is no fixed direction for the orientation of the L198 and helix 3 has a larger displacement in the results of the MD simulation. In addition, the dynamics simulation doesn’t bring about larger structural changes from mutant to wild-type structure (**Fig. 3c**; **3d**).

**Supplementary Method**

To prepare the simulation systems, the conjugate gradient minimization algorithm was applied to minimize the energy of all systems. The systems were then gradually heated from 0 to 300 K over a period of 100 ps. Following this, a 400 ps simulation was conducted to ensure that the water box of each system had an appropriate density in an isothermal-isovolumetric (NVT) ensemble. Equilibration of the systems was achieved using a 500 ps isothermal-isobaric (NPT) ensemble. During the simulations, periodic boundary conditions were applied to all systems. Bonds containing hydrogen were restrained using the SHAKE algorithm, and a 2 fs timestep was used. Temperature and pressure were controlled using weak coupling to an external bath. Long-range electrostatic interactions were calculated using the Particle Mesh Ewald method with a cutoff of 10.0 Å.

In this study, the system threshold energy was set as E = Vmax for all GaMD simulations. The maximum, minimum, average, and standard deviation values of the system potential (Vmax, Vmin, Vav, and σV) were calculated from the initial 2 ns conventional molecular dynamics. For more appropriate acceleration, the greatest acceleration parameters were determined through a short testing simulation, which was necessary to ensure that the potential statistics and GaMD acceleration parameters leveled off and achieved an optimal acceleration before running the final production simulation. Therefore, a 50 ns equilibration run was carried out in every GaMD simulation, followed by a 1s production simulation, and the simulation frames were saved every 10 ps for analysis.

**Supplementary Table**

**Table S1.** **Intra- and inter-helical distances between residues from α2 and α3 helices.** PDB structural data are average distances and standard deviations based on Å calculated for the ensemble of structures reported in the PDB. AF2 structural data are measured distances based on Å for the top model predicted by AF2. MD data are average distances based on Å from structures within 20ns after simulation stabilizing.

| PrPC | V189(Cγ1)-I184(Cδ1) | V176(Cγ1)-V180(Cγ2) | V176(Cγ1)-Y218(Cε1) |
| --- | --- | --- | --- |
| WT (PDB) | 3.8 | 4.0 | 8.3 |
| V210I (PDB) | 10.8 | 7.7 | 4.9 |
| V210I (AF2) | 4.7 | 4.1 | 9.0 |
| V210I (*MoDAFold*) | 10.5 | 8.0 | 5.4 |

**Table S2.** **Distances between residues involved in the interface of β2, α2, and α3 secondary structure elements.** PDB structural data are average distances and standard deviations based on Å calculated for the ensemble of structures reported in the PDB. AF2 structural data are measured distances based on Å for the top model predicted by AF2. MD data are average distances based on Å from structures within 20ns after simulation stabilizing.

| PrPC | Y169(Cζ)-F175(Cζ) | F175(Cζ)-Y218(Cε1) | Y163(Cε2)-Y218(Cδ1) | Y163(Cε2)- F175(Cζ) |
| --- | --- | --- | --- | --- |
| WT (PDB) | 6.4 | 4.7 | 6.4 | 5.2 |
| V210I (PDB) | 13.1 | 7.8 | 13.6 | 9.7 |
| V210I (AF2) | 5.4 | 7.3 | 10.3 | 4.4 |
| V210I (*MoDAFold*) | 13.1 | 7.2 | 14.0 | 10.0 |

**Table S3**. **Missense single-nucleotide variants selected for evaluation of MoDAFold strategy**. This table lists the 54 missense single-nucleotide variants that were identified from over 16,000 papers related to missense mutant proteins. These variants were used to select the 14 pairs of proteins with experimentally solved structures for both wild-type and mutant proteins in PDB, which were further narrowed down to the final six pairs of proteins used for evaluation of the MoDAFold strategy.

| PMID | Protein Name | Mutation Sites | | |
| --- | --- | --- | --- | --- |
|  |  | Primitive Amino Acids | Sites | Mutant Amino Acids |
| 32423068 | Gb98 | T | 251 | I |
| 32423068 | Gb98 | L | 20 | A |
| 32423068 | Ga98 | L | 45 | Y |
| 29569365 | Engrailed | L | 16 | A |
| 21839748 | prion protein | V | 210 | I |
| 21839748 | prion protein | V | 210 | I |
| 21957246 | prion protein | D | 167 | S |
| 29569365 | Engrailed | H | 94 | C |
| 11800559 | Zif268 | D | 20 | A |
| 32468185 | hemoglobin | E | 121 | Q |
| 32468185 | hemoglobin | E | 26 | K |
| 32753316 | human γS-crystallin | G | 18 | V |
| 31615672 | luciferase | S | 284 | T |
| 29993032 | Sucrose Phosphorylases | Q | 345 | F |
| 24508575 | Clp1p | G | 135 | R |
| 21138418 | aquaporins | G | 103 | D |
| 30912264 | OsCIPK7 | A | 169 | V |
| 30912264 | OsCIPK7 | R | 141 | Q |
| 34299222 | FMS-like tyrosine kinase 3 | D | 835 | Y |
| 34299222 | FMS-like tyrosine kinase 3 | D | 835 | V |
| 34299222 | FMS-like tyrosine kinase 3 | D | 835 | N |
| 34299222 | FMS-like tyrosine kinase 3 | D | 835 | I |
| 34299222 | FMS-like tyrosine kinase 3 | D | 835 | H |
| 34299222 | FMS-like tyrosine kinase 3 | D | 835 | G |
| 34299222 | FMS-like tyrosine kinase 3 | D | 835 | F |
| 34299222 | FMS-like tyrosine kinase 3 | D | 835 | E |
| 34299222 | FMS-like tyrosine kinase 3 | D | 835 | A |
| 28190170 | LipR1 | S | 130 | T |
| 22934938 | Vibrio cholerae cytolysin | A | 425 | V |
| 30798155 | TRPC3 | G | 562 | A |
| 26358403 | MeuTXKα3 | P | 30 | N |
| 25456816 | glycosylasparaginase | G | 172 | D |
| 22067166 | α-Synuclein | A | 53 | T |
| 18031265 | lysozyme | W | 62 | G |
| 17341138 | Syntaxin 1A | T | 254 | I |
| 32786304 | Huntingtin-Exon1 | - | - | - |
| 35053203 | Cytochrome P450 OleP | S | 240 | Y |
| 35053203 | Cytochrome P450 OleP | G | 92 | W |
| 35053203 | Cytochrome P450 OleP | E | 89 | Y |
| 18308323 | lamin A | E | 358 | K |
| 23426617 | Human Glucocorticoid Receptor | V | 423 | A |
| 32663558 | Glutathione S-transferases | F | 136 | A |
| 21264483 | human galactokinase enzyme | P | 28 | T |
| 28813011 | p53 | G | 245 | S |
| 30336980 | prion protein | V | 210 | K |
| 16098518 | LuxA | E | 175 | G |
| 22258055 | transglutaminase 1 | R | 142 | C |
| 17923670 | human γD-crystallin | P | 23 | T |
| 21957246 | prion protein | T | 183 | A |
| 21957246 | prion protein | N | 174 | T |
| 21957246 | prion protein | S | 170 | N |
| 14580198 | Homeobox protein VND | A | 35 | T |
| 25456816 | glycosylasparaginase | T | 203 | I |
| 18586378 | GTPase effector domain | I | 697 | A |

**Supplementary Figure**


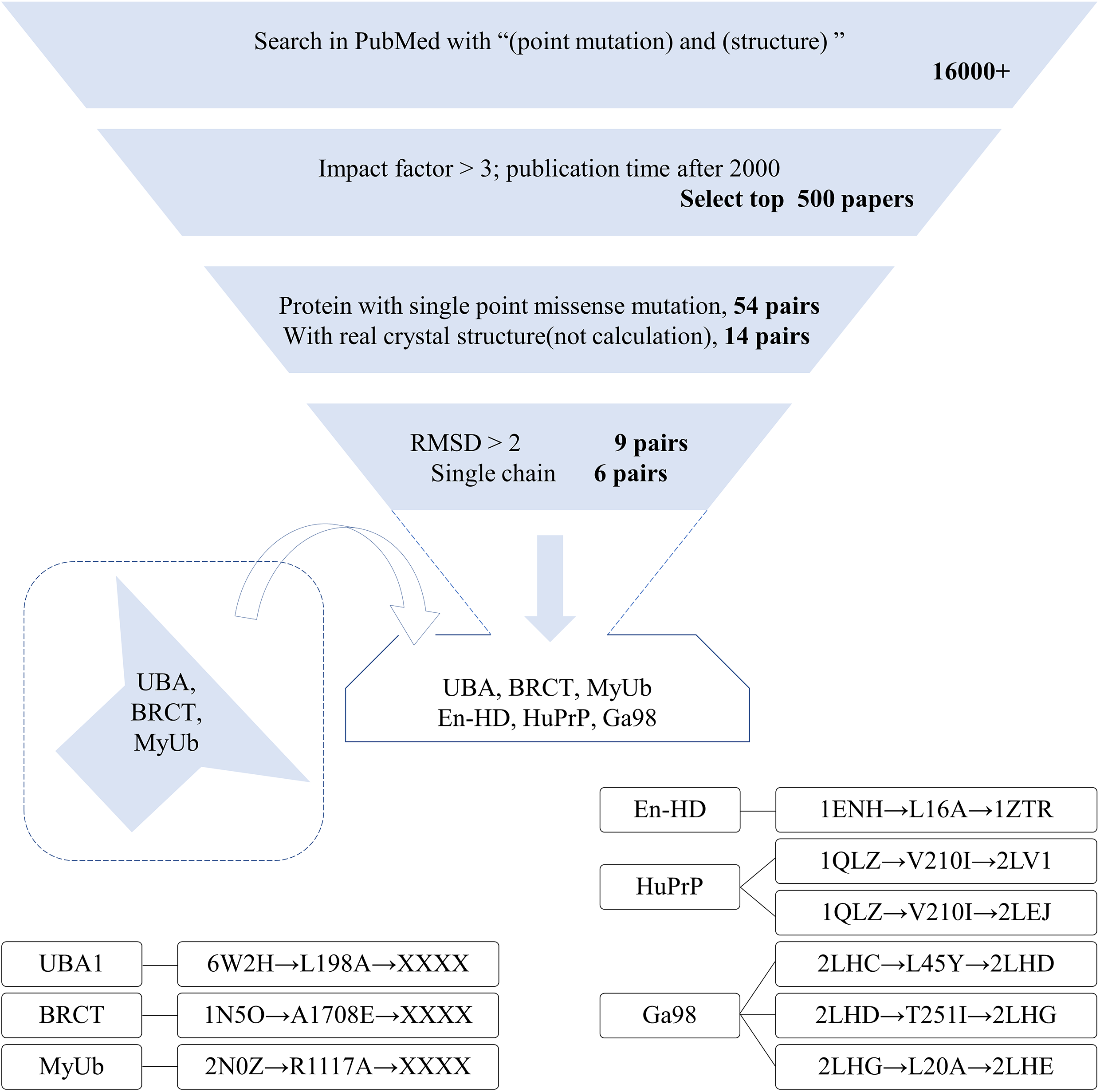


**Fig. S1. Pipeline for selection of mutant protein structures for evaluation of MoDAFold strategy**. This figure illustrates the stepwise approach used to select the mutant protein structures for the evaluation of the MoDAFold strategy. Over 16,000 papers related to missense mutant proteins were screened, and 54 missense single-nucleotide variants were identified. Fourteen pairs of proteins with experimentally solved structures for both wild-type and mutant proteins in PDB were selected, and three pairs of proteins (En-HD, HuPrP & GA98) were chosen for prediction to compare the prediction effects of different methods.


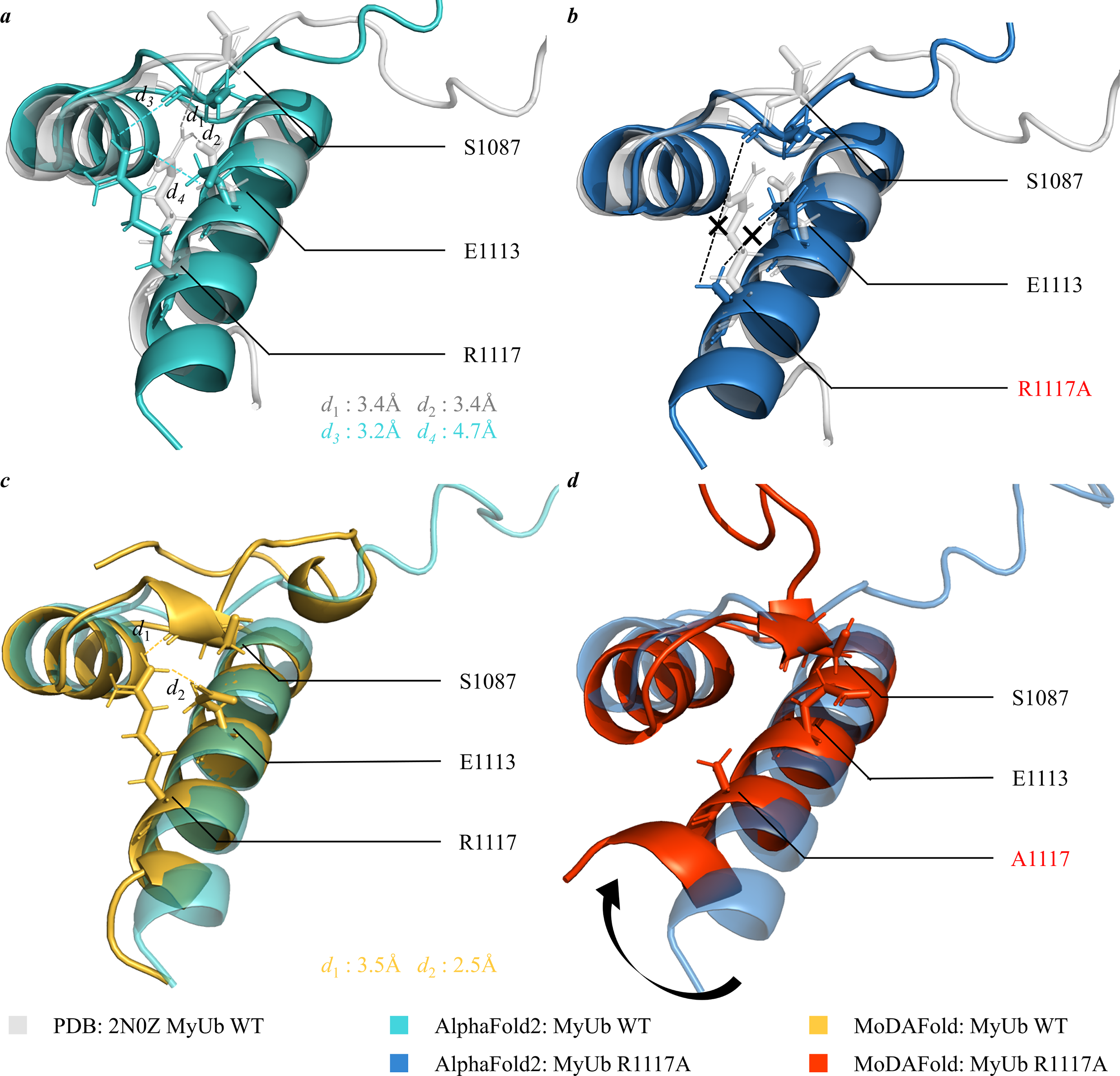


**Fig. S2.** **Structural prediction for mutant and wild-type MyUb by AlphaFold2 and *MoDAFold***. a, Overlayed the experimental MyUb structure as solved by nuclear magnetic resonance (NMR) spectroscopy (grey) and AphaFold2 predicted structure for WT MyUb (light blue). R1117 is close enough to E1113 and S1087 to form hydrogen bonds. b, Overlayed the experimental MyUb structure as solved by nuclear magnetic resonance (NMR) spectroscopy (grey) and AlphaFold2 predicted structure for MyUb R1117A (blue). Black dashed lines indicate the disappearance of hydrogen bonds among these amino acids due to mutation. c, Overlayed AlphaFold2 predicted structure (light blue) and *MoDAFold* simulated structure (yellow) for WT MyUb. R1117 is still close enough to E1113 and S1087 to form hydrogen bonds. d, Overlayed AlphaFold2 predicted structure (blue) and *MoDAFold* (orange) simulated structure for MyUb R1117A. Arrow indicates shifting of the A1117-containing helix before and after dynamics simulation.


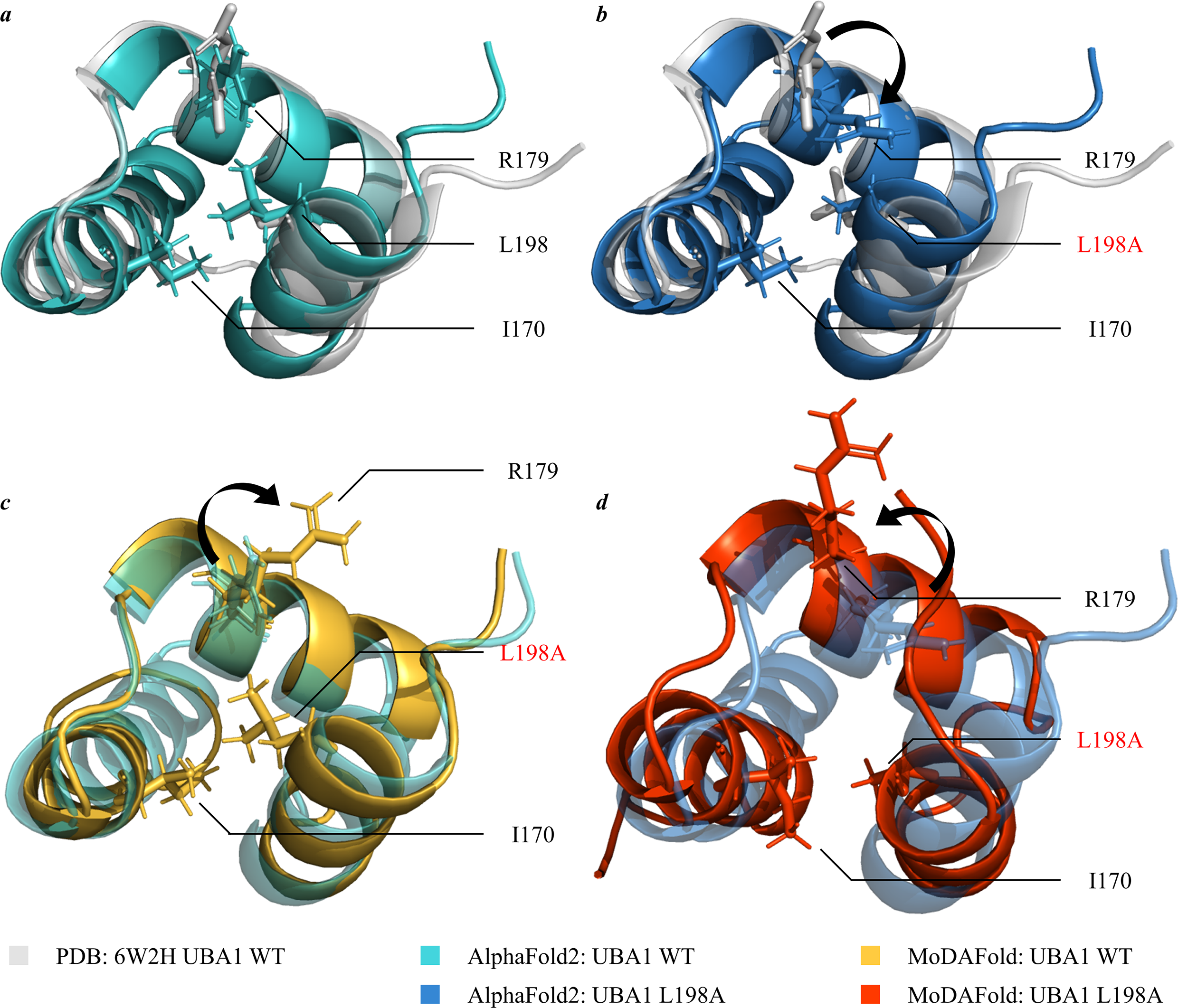


**Fig. S3.** **Structural prediction for mutant and wild-type UBA1 by AlphaFold2 and *MoDAFold***. a, Overlayed the experimental UBA1 structure (grey) and AphaFold2 predicted structure for WT UBA1 (light blue). Sidechain-heavy atoms are displayed for position 198 and the surrounding residues. b, Overlayed the experimental UBA1 structure (grey) and AphaFold2 predicted structure for UBA1 R1117 (blue). Arrow indicates R179 moving towards the mutated amino acid (L198A). c, Overlayed AlphaFold2 predicted structure (light blue) and *MoDAFold* simulated structure (yellow) for WT UBA1. Arrow indicates the rotation of R179. d, Overlayed AlphaFold2 predicted structure (blue) and *MoDAFold* simulated structure (orange) for UBA1 L198A. Arrow indicates the rotation of R179.

**Supplementary Reference**

1. Qiu Y, Wang J, Li H et al. Emerging views of OPTN (optineurin) function in the autophagic process associated with disease, Autophagy 2022;18:73-85.

2. Hu S, Wang Y, Gong Y et al. Mechanistic insights into recognitions of ubiquitin and myosin VI by autophagy receptor TAX1BP1, J Mol Biol 2018;430:3283-3296.

3. He F, Wollscheid HP, Nowicka U et al. Myosin VI contains a compact structural motif that binds to ubiquitin chains, Cell Rep 2016;14:2683-2694.

4. Biancospino M, Buel GR, Niño CA et al. Clathrin light chain A drives selective myosin VI recruitment to clathrin-coated pits under membrane tension, Nat Commun 2019;10:4974.

5. Lu XJ. DSSR-enabled innovative schematics of 3D nucleic acid structures with PyMOL, Nucleic Acids Res 2020;48:e74.

6. Ferrada MA, Savic S, Cardona DO et al. Translation of cytoplasmic UBA1 contributes to VEXAS syndrome pathogenesis, Blood 2022;140:1496-1506.

7. Tsuchida N, Kunishita Y, Uchiyama Y et al. Pathogenic UBA1 variants associated with VEXAS syndrome in Japanese patients with relapsing polychondritis, Ann Rheum Dis 2021;80:1057-1061.

8. Byeon IL, Calero G, Wu Y et al. Structure of HIV-1 Vpr in complex with the human nucleotide excision repair protein hHR23A, Nat Commun 2021;12:6864.

9. Yasuda S, Tsuchiya H, Kaiho A et al. Stress- and ubiquitylation-dependent phase separation of the proteasome, Nature 2020;578:296-300.
